# Novel detection of stem cell niche within the stroma of limbus in the rabbit during postnatal development

**DOI:** 10.1038/s41598-022-18090-2

**Published:** 2022-08-12

**Authors:** Nada Abdellah, Sara M. M. El- Desoky

**Affiliations:** 1 Department of Histology, Faculty of Veterinary Medicine, Sohag University, Sohag, 82524 Egypt; 2 Department of Anatomy and Embryology, Faculty of Veterinary Medicine, Assiut University, Asyut, 71526 Egypt

**Keywords:** Cell biology, Developmental biology, Stem cells, Structural biology

## Abstract

Identifying and locating stem cell populations in the limbus may lead to developing a cell-based strategy for treating the corneal injury. Therefore, this study was the first to design a follow-up on the microscopical and histomorphometric changes in the rabbit limbus and to localize and demonstrate the limbal stem cell niche during postnatal development. The paraffin sections from the eyes of different postnatal-developmental stages were stained and examined using light microscopy. Furthermore, sections were immunohistologically stained for the epithelial stem cell differentiation marker, cytokeratin-14. Moreover, semithin and ultrathin sections were applied for ultrastructural demonstration of the stem cell niche. This study revealed that the number and thickness of limbal epithelial layers increased with age, whereas the thickness of limbal stroma decreased. Additionally, the immunohistochemical data showed that ck14 staining intensity increased in the limbal region where limbal stem cells reside. The semithin and ultrastructure investigation revealed stem cell clusters within the limbus’s underlying stroma close to the blood and nerve supply and surrounded by telocytes. Conclusively, isolated clusters of limbal epithelial stem cells combined with blood vessels, nerve fibers, and telocytes propose a harmonious microenvironment of a stem cell niche.

## Introduction

The limbus is the transitive area between the transparent cornea and opaque sclera. It consists of the superficial epithelial layer and the underlying connective tissue^1,2^. Interestingly, the limbus is differentiated from the cornea under the light microscope using its lack of an orderly arrangement in the collagen fibrils and blood vessels^3^. Although the cornea and the limbus possess similar epithelium, the limbal epithelium comprises numerous epithelial dendritic cells, T lymphocytes, and highly pigmented melanocytes. Notably, the basal epithelial cells are the least differentiated cells of the ocular surface epithelium. These cells are smaller, less columnar, and possess several cytoplasmic organelles^4^. Previous studies have supported the opinion that these cells are limbal epithelial stem cells (LESCs), which produce many differentiated corneal epithelia, providing excellent therapeutic prospects^4–7^. This finding explains why most surveys have dealt exclusively with the limbal epithelium to provide insight into the attempt to repair the superficial corneal damage caused by limbus transplantation^5,8,9^. Although several positive markers for limbal epithelial stem cells (LESC) have been obtained, these markers still conflict in specificity and alter the differentiating stem from progenitor and early differentiated cells^10^. Keratins (K-14, K-15, and K-19), integrins and adhesion molecules (a6, avb5, and N-cadherin), transcription factors (DNp63a and C/EBPd), and membrane transporter proteins (ABCG2) are considered as extensively accepted positive identifiers^11,12^. Although ABCG2 seems to be the best suitable cell surface marker for identifying and isolating corneal epithelial SC, it reduced to small clusters of basal cells in the limbal epithelium. However, K14 shows a higher expression level in basal limbal cells^11^. Keratin 14, also known as cytokeratin-14 (CK-14), is a type I acidic keratin expressed in mitotically active basal cells of the stratified epithelium^13,14^. The lineage-tracing production of fluorescent proteins driven by a K14 promoter has recently followed the limbal stem cell-driven migration. All fluorescent cells following induction will be the progeny of those progenitors since this gene is restricted to stem and progenitor cells in the limbus^14,15^. Moreover, the recognized LESCs of basal limbal epithelial cells, a research group detected solid cords of epithelial cells within the underlying stroma of the limbus termed the limbal epithelial crypt (LEC). These cells express both ABCG-2 and CK14 stem cell markers, and the LEC is expected to make up a putative stem cell niche^16,17^. Although the limbus was proposed as the site of epithelial stem cells of the cornea, no histological stem cell niche has been detected in offspring rabbits. Therefore, this study localized and demonstrated the limbal stem cell niche during a postnatal developmental study in rabbit limbus.

## Materials and methods

### Sample collection

Clinically normal New Zealand white rabbit neonates five animals for each group were randomly collected from a farm of the Faculty of Agriculture, Assiut University, Egypt. Regardless of sex, animals were categorized into four different age groups at 1, 7, 15, and 30 postnatal days (PNDs). At PND30, the eyelids were fully opened, and the eye was functionally active. All methods in the present study were carried out in accordance with relevant guidelines and regulations. The Faculty of Veterinary Medicine National Ethical Committee, Assiut University, Egypt, has authorized all the steps in the present work. All methods were reported in accordance with the ARRIVE guidelines (https://arriveguidelines.org).

### Tissue preparing

The animals from each group were anesthetized using sevoflurane (US Pharmacopeia) as an inhalational agent and were subsequently euthanized by exsanguination through the abdominal aorta. After euthanasia, the eyes and eyelids for each group were removed through enucleation and were immediately fixed in 10% neutral buffered formalin overnight at 4 °C. After washing, the fixed samples were dehydrated in ascending grades of ethanol, cleared in xylene, and impregnated with melted paraffin wax (Sigma-Aldrich, USA). Finally, paraffin blocks of the processed samples were prepared. The thin sections (5–6 µm thick) were cut using a Richert Leica RM 2125 Microtome, Germany, and mounted on glass slides. The sections were stained with Harris’s Hematoxylin, Eosin, (H&E), and Crossmon’s Trichrome^18^.

### Histomorphometric measurements

The limbus epithelium’s and stroma’s thicknesses were estimated using ImageJ version 1.48 software. The parameters were measured using five non-overlapping fields from four different sections of each animal.

### Statistical analysis

Data on the histomorphometric measurements were expressed as mean ± S.D. After that, measurements were statistically estimated via GraphPad Prism (version 5) (San Diego, California, USA) using one-way ANOVA with Tukey’s post hoc test. A *P* value of *P* < 0.05 was considered statistically significant.

### Immunohistochemistry

The sections were deparaffinized with xylene, hydrated with a descending grade of ethanol, and then washed with 0.1-M PBS (3 × 10 min). Next, the antigen retrieval was conducted to decrease the masking of antigen epitopes, using 0.1-M sodium citrate buffer solution (pH = 6) for 7 min using a microwave (600 W). Subsequently, the sections were cooled to room temperature (RT) for 20 min and washed with PBS (pH 7.4) for 10 min. After blocking the endogenous peroxidase activity with 3% H2O2 in H2O for 30 min at RT, the sections were washed with PBS (3 × 5 min), then the sections were blocked with 10% normal donkey serum (NDS) + 0.2% Triton-X100/PBS for 2 h at RT. Subsequently, sections were incubated overnight at 4 °C with the following antibody mouse anti-cytokeratin 14 (LL001, Santa Cruz Biotechnology; 1:300). The sections were rinsed 3 × 10 min in 0.2% Triton-X 100/PBS and incubated with donkey antimouse IgG-B (Santa Cruz Biotechnology; 1:200) for 2 h at RT, followed by incubation with vectastain ABC (Avidin–Biotin complex) reagent for 45 min in a humid chamber at RT. Visualization of the reaction was conducted with DAB for 5–10 min. The sections were counterstained with Harris hematoxylin for 30 s. Finally, the sections were dehydrated in a graded series of ethanol, cleared with xylene, and covered with mounting media called DPX (Dibutyl phthalate Polystyrene Xylene). Immunohistochemical staining was evaluated using a Leitz Dialux 20 Microscope, and images were photographed using a Canon digital camera (Canon Powershot A95).

### Semithin sections and transmission electron microscopy preparations (TEM)

Representative specimens from the cornea and limbus at PND30 were fixed in 2.5% glutaraldehyde in 0.1-M Na-cacodylate buffer, pH 7.2 for 24 h at 4 °C^19^. The samples were washed in the same buffer, then post-fixed in 1% osmic acid in a 0.1-M Na-cacodylate buffer for 2 h at RT. The samples were then dehydrated in ascending grades of ethanol and embedded in an Araldite–Epon mixture. Semi-thin sections were cut at 1 μm thickness and stained with 1% toluidine blue^20^. A Leitz Dialux 20 microscope was used to examine the stained sections. The photos were taken using a Canon digital camera (Canon Power shot A 95). Ultra-thin sections were stained with uranyl acetate and lead citrate^21^ and imaged in a JEOL 100 II transmission electron microscope (JEOL, Tokyo, Japan) at the Electron Microscopy Unit of Assiut University.

### Ethical approval and consent to participate

The study was approved by the Ethics Committee of Assiut University, Egypt.

## Results

### Postnatal development of limbus epithelium and stroma

**Figure 1:**
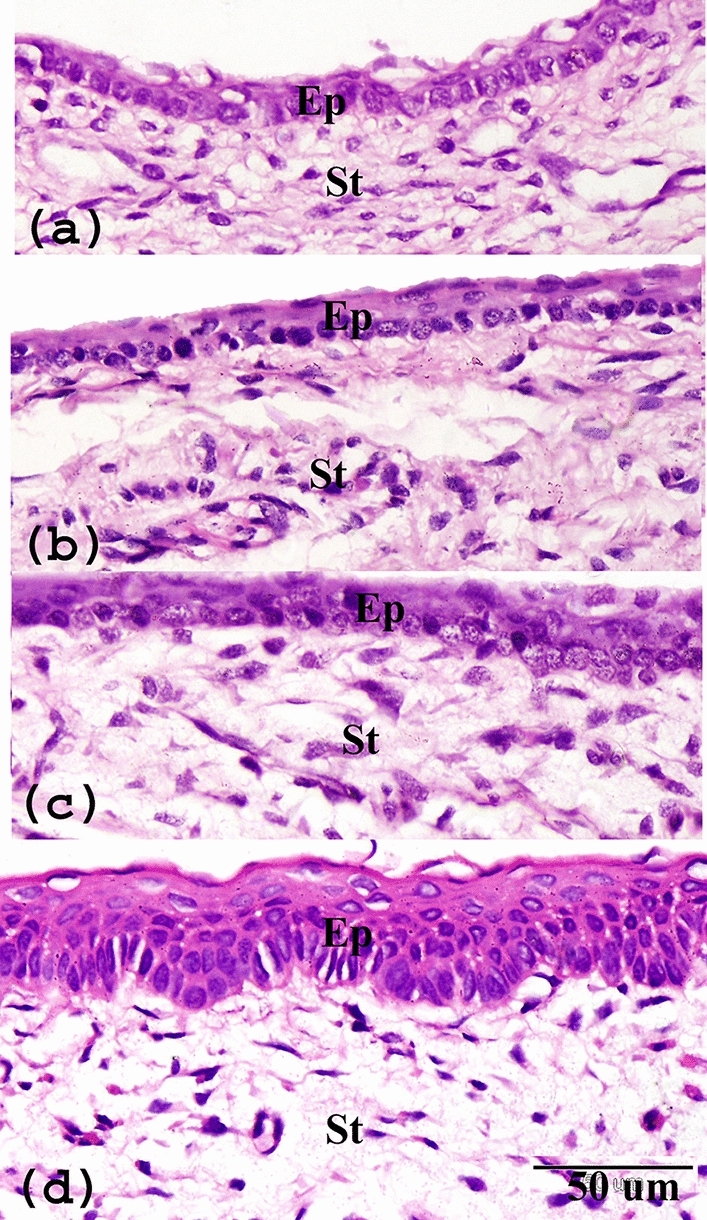
Paraffin sections stained with H&E showing postnatal development of limbal epithelium (**a**) PND1, one to two layers of cells presented in the limbal epithelium. (**b**) At PND7, slightly increased to three-four layers. (**c**) At PND15, increased to five to six layers, (**d**) At PND30, the thickness of limbal epithelium was highly differentiated and showed mature stratified squamous epithelium. Note epithelium (Ep) and stroma (St).

**Figure 2:**
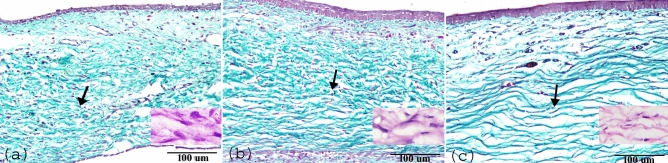
Paraffin sections showing postnatal development of limbal stroma stained with Crossmon's Trichrome (**a**) At PND7, (**b**) PND15, and (**c**) at PND30 showing the collagen fibers in the inner fibrous layer were more orderly organized with age (black arrow). Note the shape of the keratocytes nucleus transformed from oval to flat (H&E staining).

**Figure 3:**
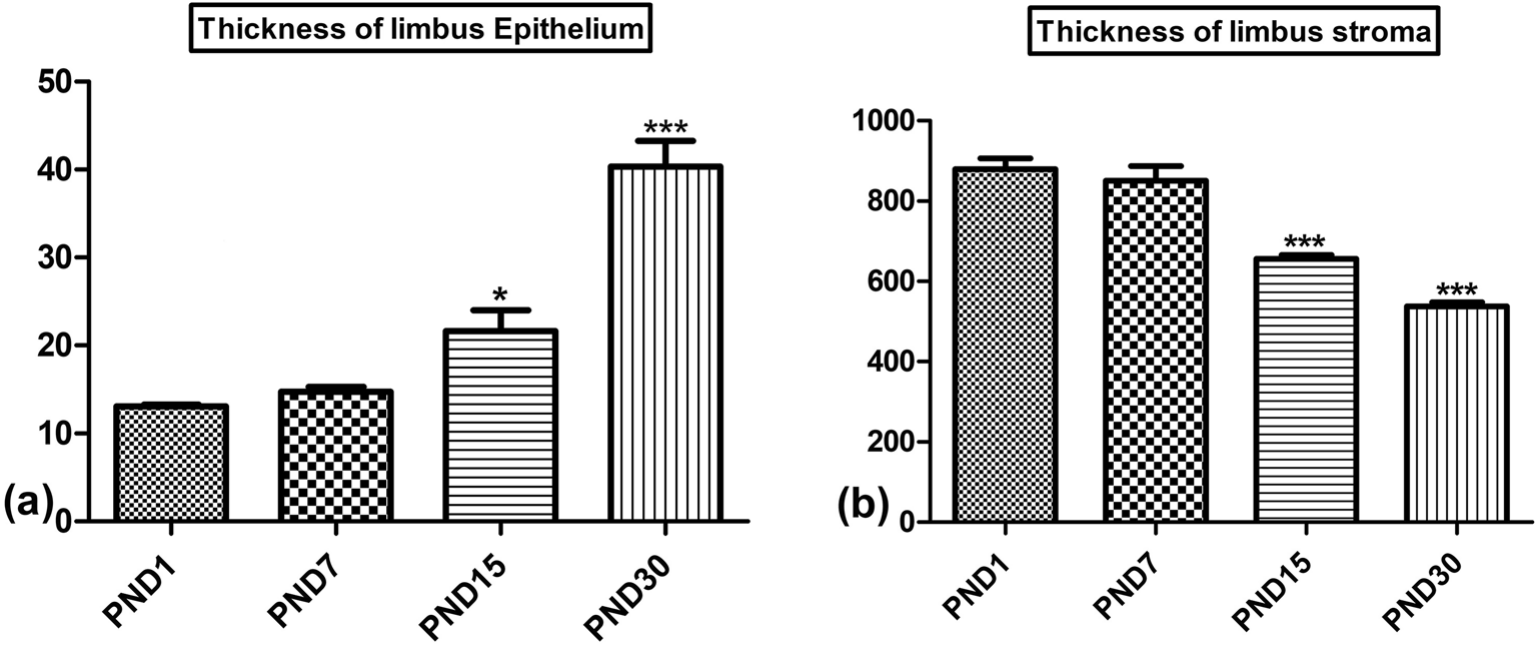
Morphomerical measurements showing postnatal development of limbus. (**a**) The thickness of limbal epithelium significantly increased on PND15 and PND30. (**b**) The thickness of limbal stroma significantly decreased on PND15 and PND30. Significant differences versus the PND1 determined through one-way ANOVA with Tukey’s post hoc test are marked by different asterisks: *P ≤ 0.05, **P ≤ 0.01, ***P ≤ 0.001).

This study revealed a remarkable limbal epithelium differentiation and development from PND1 to PNDs30. On PND1, one to two layers of cells were presented in the limbal epithelium. The limbal epithelium was composed of cuboidal basal cells with superficial layers of flattened cells (Fig. 1a). At PND7, the thickness of the limbal epithelium slightly increased and showed about three-four layers (Fig. 1b). At PND15, the limbal epithelial increased to five to six layers (Fig. 1c). The thickness of the limbal epithelium extended to seven to eight layers at PND30 and the epithelium showed prominent stratified squamous epithelium (Fig. 1d). The thickness of the limbal epithelium significantly increased from 13.05 ± 0.5 µm on PND1 to 42.9 ± 3.9 µm on PND30 (Fig. 3a). Additionally, the stroma of the limbus exhibited observable changes. The limbal stroma comprised a surface fibroareolar layer, which contains limited stromal connective tissue with the blood vessels and stromal keratocytes, including an inner fibrous layer containing dense collagen fibers with dense stromal keratocytes. The collagen fibers in the inner fibrous layer were more orderly arranged with age, and the shape of the nucleus of stromal keratocytes changed from oval to flat (Fig. 2a–c). Unlike the limbal epithelium, the stromal thickness significantly decreased from 880 ± 33 µm on PND1 to 538.6 ± 12.5 µm on PND30 (Fig. 3b).

### Immunohistochemistry

**Figure 4:**
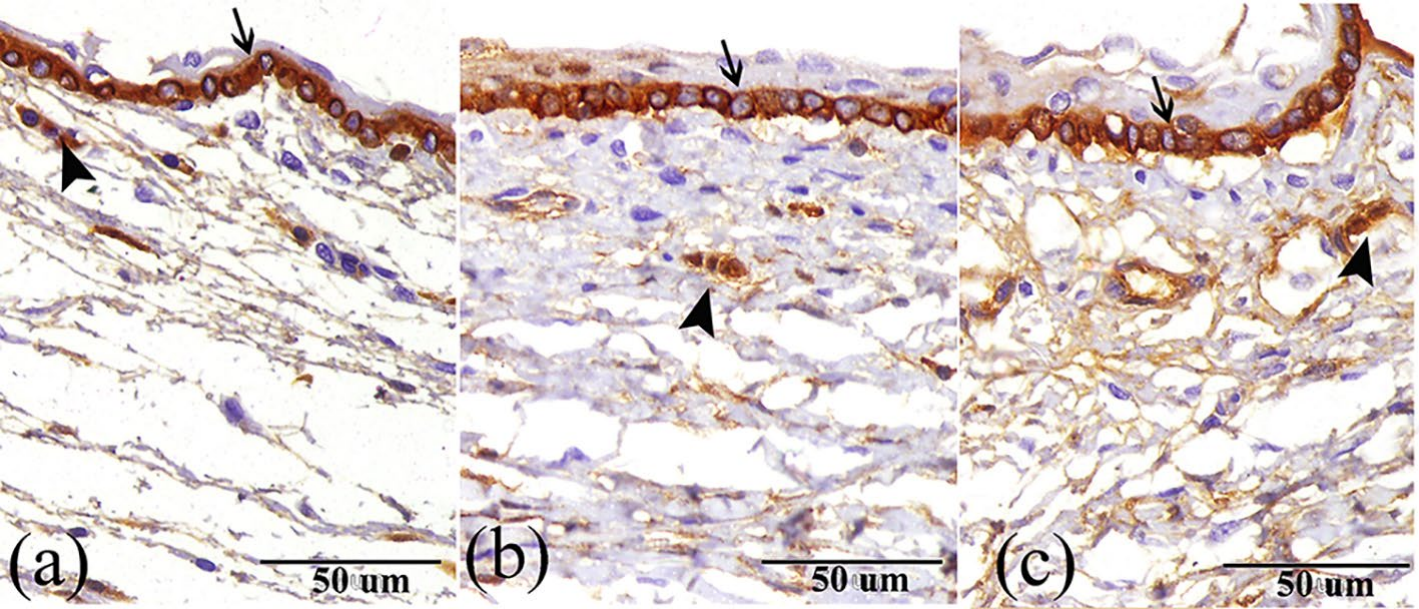
Immunohistochemical staining of the limbus to ck14 (**a**) At PND7, (**b**) At PND15, and (**c**) At PND30 showing positive immunoreactivity of the basal layer of the limbal epithelium (arrow) and the limbal epithelial stem cells clusters located within the underlaying stroma (arrowhead).

The immunohistochemical data of PND7 to PND30 revealed that the basal layer of the limbal epithelial cells was highly expressed ck14 (Fig. 4a–c), and the limbal epithelial cell clusters located within the underlying stroma were positive proposing where limbal stem cells are located (Fig. 4a–c).

### Semithin sections

**Figure 5:**
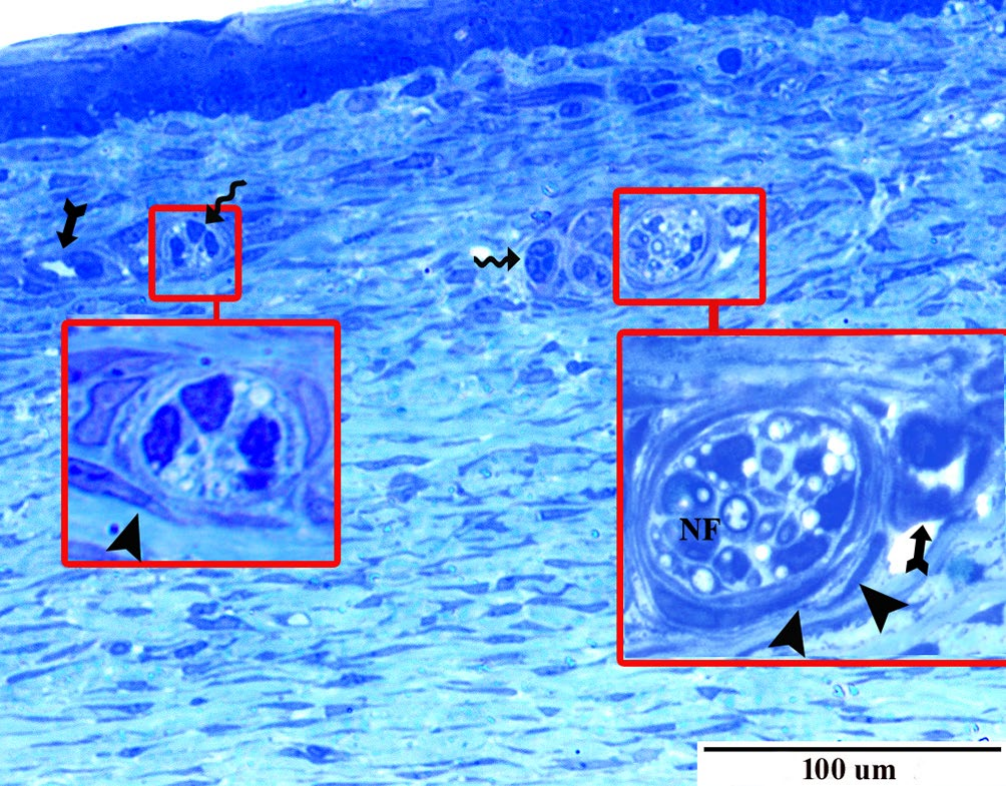
Semi-thin section of PND30 stained with toluidine blue showing a presence of epithelial cell clusters within the stroma of the limbus (wavy arrow) located nearby blood vessels (forked arrow) and nerve fibers (NF) and surrounded by telocytes (arrowhead).

Investigation of semithin sections of PND30 revealed the presence of epithelial cellular aggregation within the underlying stroma of the limbus forming clusters. These clusters were located nearby blood and nerve supply and were surrounded by cells with telocytes proposing a stem cell niche (Fig. 5).

### TEM of limbal epithelial stem cells and stem cell niche

**Figure 6:**
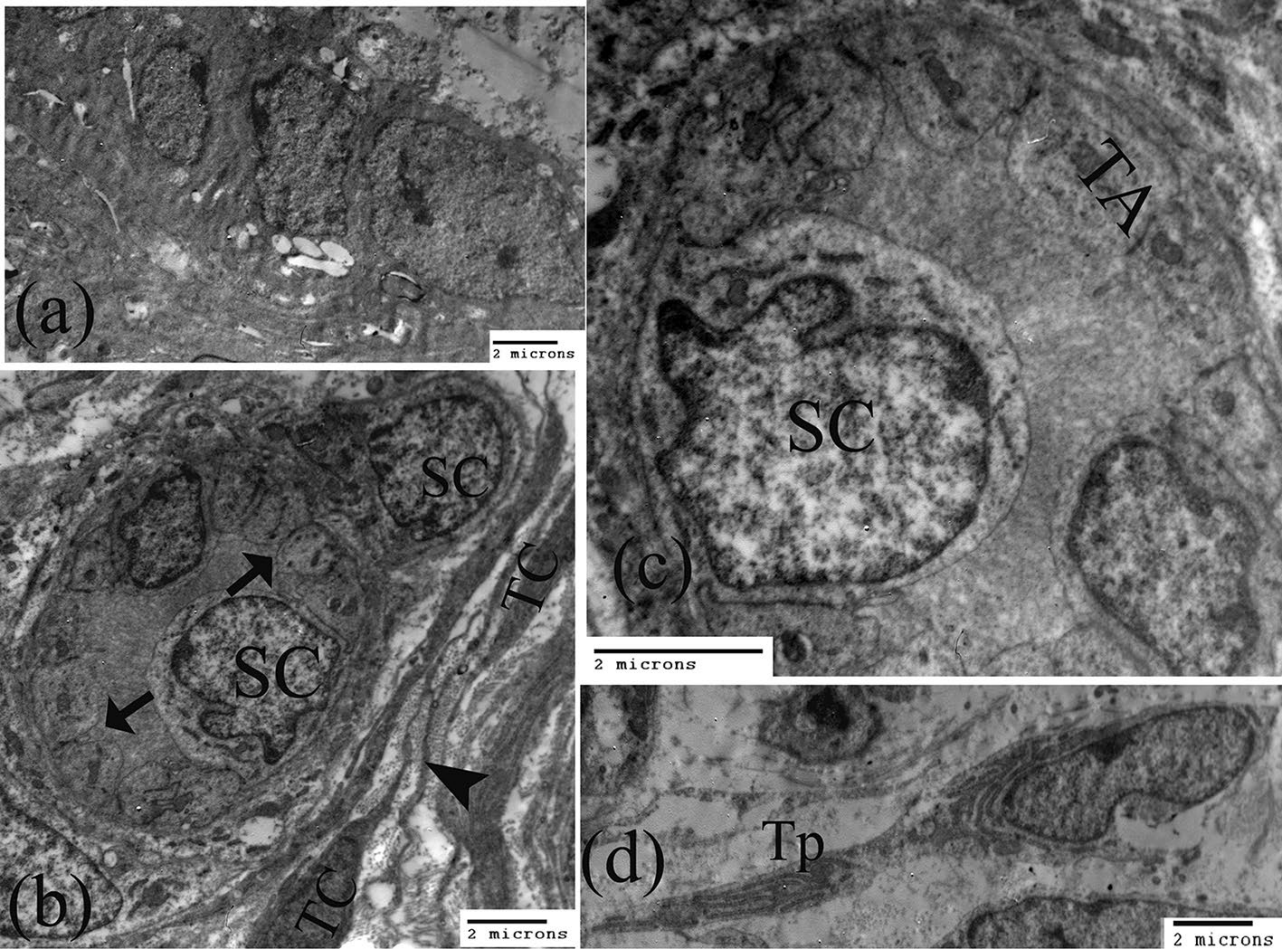
Transmission electron microscopy images showing (**a**) The basal limbal stem cells (LESCs), and (**b, c**) The limbal stromal epithelial stem cells clusters presented two types of cells, putative stem cells (SC) large cells characterized by high nuclear to cytoplasmic ratio with a large, indented nucleus, and populations of the transit-amplifying epithelial cells (TA)(arrow) small basal cuboidal cells hold a large nucleus with a prominent nucleolus. The limbal stromal epithelial stem cells cluster was enclosed by telocytes (TC) with long telopods (arrowhead). (**d**) The telocytes (TC) contain an indented oval nucleus bounded by a thin layer of cytoplasm and have long telepods (Tp) packed with rER.

The basal limbal stem cells (LESCs) were morphologically composed of orderly arranged cuboidal cells containing a large irregular-shaped euchromatic nucleus (Fig. 6a). The stem cell niches were demonstrated to include the limbal epithelial stem cell clusters located in the underlying stroma which showed two types of cells, putative stem cells, which were large cells characterized by high nuclear to cytoplasmic ratio, and the thin cytoplasm that possessed rough endoplasmic reticulum (rER) and mitochondria. The nucleus was large, indented, and contain peripheral heterochromatin. Additionally, populations of small basal cuboidal cells were demonstrated to contain a large nucleus with prominent nucleolus and euchromatin, proposing the transit-amplifying epithelial cells (TAs) (Fig. 6b,c).

Moreover, telocytes could be identified surrounding the limbal epithelial stem cell clusters (Fig. 6b,c). The telocytes (TCs) exhibited an indented oval nucleus surrounded by a thin layer of cytoplasm and long cellular processes named telepods (Tp). Additionally, the telepods showed well-developed rough endoplasmic reticulum (Fig. 6d).

## Discussion

In this study, we examined the postnatal histomorphological development of the limbus of the eye in New Zealand white rabbits from immediately after birth to PND30. The limbal epithelial histological changes align with the corneal epithelial changes reported by Yamagiwa and colleagues^22^. Additionally, the limbal epithelial thickness increased with age, which is similar to the morphometrical findings illustrated in mice^23^. Although the morphological changes of the limbal stroma correspond with the corneal one, the thickness of the limbal stroma did not increase with age but decreased^22,24^. However, there is no specific antibody to detect stem cells in the limbus. Furthermore, ck14 has been previously used as a marker for limbus basal epithelial differentiation and SC characterization in humans and equines^17,25^. Therefore, our immunohistochemistry staining for ck14 detected a positive reaction at the basal layer of the limbal epithelium and in the limbal epithelial stem cell clusters situated within the underlying stroma. We conducted further semithin and ultrastructure evaluation at PND30 since the limbus was histologically developed like that of an adult^22^. According to the immunohistochemical observation, we detected a novel histological structure consisting of epithelial cell clusters in the semithin sections. Furthermore, we found evidence for the stem cell niche in the limbus in the rabbit using TEM. Previous human studies have identified the anatomical structure named limbal epithelial crypts as a stem cell niche. These previous studies revealed that these limbal epithelial crypts develop from the limbal palisades of Vogt^16,17,26^. However, it varies in the rabbit limbal epithelium. Additionally, the previous findings confirmed the absence of the limbal palisades of Vogt; however, there are lobed protrusions from the basal epithelial cells to the underlying stroma that may function as the palisades^2,27,28^. Additionally, Yamada and associates demonstrated an interaction between the limbal basal epithelial cells and the underlying stromal mesenchymal cells as a hypothetical stem cell niche in rabbits^28^. Our observation revealed that the cluster of epithelial cells in underlying limbal stroma located beside blood vessels to support nutrient supply agrees with the limbal palisades in humans^29^. Furthermore, we detected nerve endings near the stromal epithelial cell clusters similar to limbal corpuscular nerve endings demonstrated in humans between the palisades^30^. Our TEM showed different types of cells within the clusters. We demonstrated large cells with a high nucleus to cytoplasmic ratio characteristic of putative stem cells^28,29,31^. Additionally, we identified populations of small cells that contain nuclei with more euchromatin and prominent nucleoli, which agree with transient amplifying cells demonstrated early in 1993^11,32^. Moreover, our observation revealed that telocytes surrounded the epithelial cell clusters. Previously, the ultrastructure of telocytes was demonstrated clearly as stromal cells^31,33–35^. Telocytes, blood vessels, nerve ending, and limbal epithelial stem cells were isolated in clusters. Our finding aligns with a previous study on the eye of a mouse that was defined as stem-cell niches^31^. A study by Cantarero and associations hypothesized that blood vessels and nerve fibers might initiate a compatible microenvironment in terms of (“niche”) in the differentiation process of mesenchymal precursor cells, which aligns with our observation. Consequently, the telocytes could produce exosomes to regulate their microenvironment^36^.

## Conclusion

This study revealed that the number of limbal epithelial layers and thickness increased with age. However, the thickness of limbal stroma decreased with age. Additionally, our investigation showed stem cell clusters of putative stem cells and transient amplifying cells within the underlying stroma of the limbus. Furthermore, the stem cell clusters were close to the blood and nerve supplies and surrounded by telocytes, signifying a harmonious microenvironment for the stem cell niche.

## Data Availability

The datasets generated and analyzed during this study are available from the corresponding author on request.
